# Supplementary material for: Unveiling the Bioactive Potential of the Invasive Jellyfish Phyllorhiza punctata Through Integrative Transcriptomic and Proteomic Analyses
Source: Biomolecules. 2025 Aug 4;15(8):1121. doi: 10.3390/biom15081121 (PMC12383608; doi:10.3390/biom15081121)
Supplement: Supplementary file 1 [file biomolecules-15-01121-s001.zip › Suplementary Material/FileS3/PEPFOLD4/XP_065057120_8080/clusters.html]

 PEP-FOLD clusters


Download archive of all models  here   

|  |  |  |
| --- | --- | --- |
| # | file | sOPEP |
| # cluster 1 - Size: 12 |  |  |
|  | XP\_065057120\_8080-model1 | -27.0284 |
|  | XP\_065057120\_8080-model1.1 | -27.0202 |
|  | XP\_065057120\_8080-model1.2 | -26.9986 |
|  | XP\_065057120\_8080-model1.3 | -26.9466 |
|  | XP\_065057120\_8080-model1.4 | -26.9196 |
|  | XP\_065057120\_8080-model1.5 | -26.8271 |
|  | XP\_065057120\_8080-model1.6 | -26.7418 |
|  | XP\_065057120\_8080-model1.7 | -26.3313 |
|  | XP\_065057120\_8080-model1.8 | -26.3106 |
|  | XP\_065057120\_8080-model1.9 | -26.1251 |
|  | XP\_065057120\_8080-model1.10 | -25.9433 |
|  | XP\_065057120\_8080-model1.11 | -25.9106 |
| # cluster 2 - Size: 86 |  |  |
|  | XP\_065057120\_8080-model2 | -23.5748 |
|  | XP\_065057120\_8080-model2.1 | -23.381 |
|  | XP\_065057120\_8080-model2.2 | -23.3784 |
|  | XP\_065057120\_8080-model2.3 | -23.3738 |
|  | XP\_065057120\_8080-model2.4 | -23.3451 |
|  | XP\_065057120\_8080-model2.5 | -23.3031 |
|  | XP\_065057120\_8080-model2.6 | -23.295 |
|  | XP\_065057120\_8080-model2.7 | -23.2876 |
|  | XP\_065057120\_8080-model2.8 | -23.2838 |
|  | XP\_065057120\_8080-model2.9 | -23.2826 |
|  | XP\_065057120\_8080-model2.10 | -23.2793 |
|  | XP\_065057120\_8080-model2.11 | -23.2544 |
|  | XP\_065057120\_8080-model2.12 | -23.2435 |
|  | XP\_065057120\_8080-model2.13 | -23.2309 |
|  | XP\_065057120\_8080-model2.14 | -23.2245 |
|  | XP\_065057120\_8080-model2.15 | -23.2229 |
|  | XP\_065057120\_8080-model2.16 | -23.2126 |
|  | XP\_065057120\_8080-model2.17 | -23.2121 |
|  | XP\_065057120\_8080-model2.18 | -23.197 |
|  | XP\_065057120\_8080-model2.19 | -23.196 |
|  | XP\_065057120\_8080-model2.20 | -23.1742 |
|  | XP\_065057120\_8080-model2.21 | -23.1623 |
|  | XP\_065057120\_8080-model2.22 | -23.1381 |
|  | XP\_065057120\_8080-model2.23 | -23.1367 |
|  | XP\_065057120\_8080-model2.24 | -23.1203 |
|  | XP\_065057120\_8080-model2.25 | -23.1192 |
|  | XP\_065057120\_8080-model2.26 | -23.1151 |
|  | XP\_065057120\_8080-model2.27 | -23.1067 |
|  | XP\_065057120\_8080-model2.28 | -23.1056 |
|  | XP\_065057120\_8080-model2.29 | -23.1042 |
|  | XP\_065057120\_8080-model2.30 | -23.1033 |
|  | XP\_065057120\_8080-model2.31 | -23.1026 |
|  | XP\_065057120\_8080-model2.32 | -23.1003 |
|  | XP\_065057120\_8080-model2.33 | -23.0886 |
|  | XP\_065057120\_8080-model2.34 | -23.0861 |
|  | XP\_065057120\_8080-model2.35 | -23.0787 |
|  | XP\_065057120\_8080-model2.36 | -23.0769 |
|  | XP\_065057120\_8080-model2.37 | -23.0761 |
|  | XP\_065057120\_8080-model2.38 | -23.0669 |
|  | XP\_065057120\_8080-model2.39 | -23.0655 |
|  | XP\_065057120\_8080-model2.40 | -23.0474 |
|  | XP\_065057120\_8080-model2.41 | -23.0469 |
|  | XP\_065057120\_8080-model2.42 | -23.046 |
|  | XP\_065057120\_8080-model2.43 | -23.0333 |
|  | XP\_065057120\_8080-model2.44 | -23.0264 |
|  | XP\_065057120\_8080-model2.45 | -23.0257 |
|  | XP\_065057120\_8080-model2.46 | -23.022 |
|  | XP\_065057120\_8080-model2.47 | -23.0194 |
|  | XP\_065057120\_8080-model2.48 | -23.0167 |
|  | XP\_065057120\_8080-model2.49 | -23.0165 |
|  | XP\_065057120\_8080-model2.50 | -23.0138 |
|  | XP\_065057120\_8080-model2.51 | -23.0126 |
|  | XP\_065057120\_8080-model2.52 | -23.0081 |
|  | XP\_065057120\_8080-model2.53 | -23.0081 |
|  | XP\_065057120\_8080-model2.54 | -23.0081 |
|  | XP\_065057120\_8080-model2.55 | -23.0079 |
|  | XP\_065057120\_8080-model2.56 | -23.0059 |
|  | XP\_065057120\_8080-model2.57 | -23.0051 |
|  | XP\_065057120\_8080-model2.58 | -23.0018 |
|  | XP\_065057120\_8080-model2.59 | -22.9987 |
|  | XP\_065057120\_8080-model2.60 | -22.9955 |
|  | XP\_065057120\_8080-model2.61 | -22.9908 |
|  | XP\_065057120\_8080-model2.62 | -22.9882 |
|  | XP\_065057120\_8080-model2.63 | -22.9862 |
|  | XP\_065057120\_8080-model2.64 | -22.9859 |
|  | XP\_065057120\_8080-model2.65 | -22.9833 |
|  | XP\_065057120\_8080-model2.66 | -22.9785 |
|  | XP\_065057120\_8080-model2.67 | -22.9769 |
|  | XP\_065057120\_8080-model2.68 | -22.9768 |
|  | XP\_065057120\_8080-model2.69 | -22.9742 |
|  | XP\_065057120\_8080-model2.70 | -22.9657 |
|  | XP\_065057120\_8080-model2.71 | -22.9579 |
|  | XP\_065057120\_8080-model2.72 | -22.9379 |
|  | XP\_065057120\_8080-model2.73 | -22.9353 |
|  | XP\_065057120\_8080-model2.74 | -22.9325 |
|  | XP\_065057120\_8080-model2.75 | -22.9163 |
|  | XP\_065057120\_8080-model2.76 | -22.8905 |
|  | XP\_065057120\_8080-model2.77 | -22.8809 |
|  | XP\_065057120\_8080-model2.78 | -22.8805 |
|  | XP\_065057120\_8080-model2.79 | -22.8633 |
|  | XP\_065057120\_8080-model2.80 | -22.8239 |
|  | XP\_065057120\_8080-model2.81 | -22.8171 |
|  | XP\_065057120\_8080-model2.82 | -22.7951 |
|  | XP\_065057120\_8080-model2.83 | -22.7905 |
|  | XP\_065057120\_8080-model2.84 | -22.7844 |
|  | XP\_065057120\_8080-model2.85 | -22.7658 |
| # cluster 3 - Size: 2 |  |  |
|  | XP\_065057120\_8080-model3 | -23.0966 |
|  | XP\_065057120\_8080-model3.1 | -22.9219 |
|  |  |  |
